# Adsorption and Degradation of Volatile Organic Compounds by Metal–Organic Frameworks (MOFs): A Review

**DOI:** 10.3390/ma15217727

**Published:** 2022-11-02

**Authors:** Yangyang Xie, Sining Lyu, Yue Zhang, Changhong Cai

**Affiliations:** 1 Department of Building Environment and Energy Engineering, School of Civil and Resources Engineering, University of Science and Technology Beijing, Beijing 100083, China; 2 School of Energy and Power Engineering, Xi’an Jiaotong University, Xi’an 710049, China; 3 School of Materials Science and Engineering, University of Science and Technology Beijing, Beijing 100083, China

**Keywords:** metal–organic framework, volatile organic compounds, adsorption, degradation

## Abstract

Volatile organic compounds (VOCs) are a major threat to human life and health. The technologies currently used to remove VOCs mainly include adsorption and photocatalysis. Adsorption is the most straightforward strategy, but it cannot ultimately eliminate VOCs. Due to the limited binding surface, the formaldehyde adsorption on conventional photocatalysts is limited, and the photocatalytic degradation efficiency is not high enough. By developing novel metal–organic framework (MOF) materials that can catalytically degrade VOCs at room temperature, the organic combination of new MOF materials and traditional purification equipment can be achieved to optimize adsorption and degradation performance. In the present review, based on the research on the adsorption and removal of VOCs by MOF materials in the past 10 years, starting from the structure and characteristics of MOFs, the classification of which was described in detail, the influencing factors and mechanisms in the process of adsorption and removal of VOCs were summarized. In addition, the research progress of MOF materials was summarized, and its future development in this field was prospected.

## 1. Introduction

**Figure 1:**
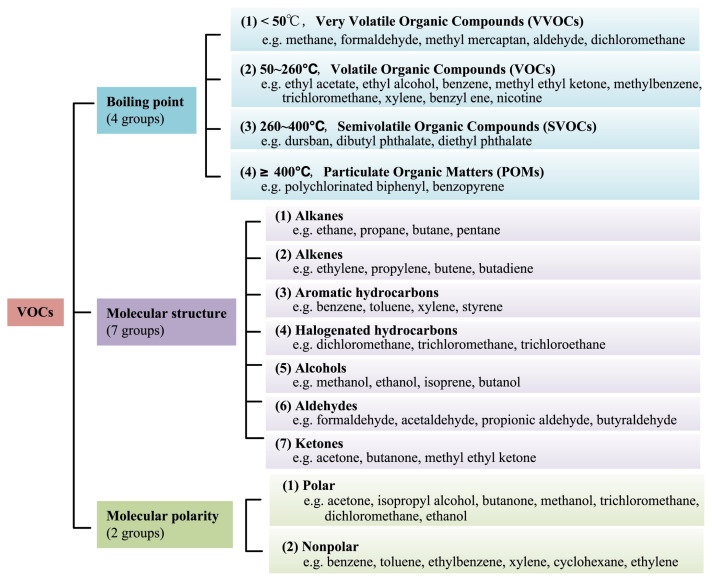
The classification of different VOCs [2].

Volatile organic compounds (VOCs) in the atmosphere are a group of organic substances characterized by a low boiling point. This is defined by the United States Environmental Protection Agency (US EPA) as any carbon compound that participates in the photochemical reaction in the atmosphere, except for carbon monoxide, carbon dioxide, carbonic acid, metal carbides or carbonates, and ammonium carbonate. The World Health Organization (WHO) considers VOCs as organic compounds with a saturated vapor pressure exceeding 133.322 Pa at atmospheric pressure and a boiling point between 50 and 260 °C [1]. WHO classifies VOCs into very volatile organic compounds (VVOCs), VOCs, semi-volatile organic compounds (SVOCs), and particulate organic compounds (POMs) according to their boiling points. The World Health Organization’s (WHO) classification of VOCs according to boiling point is shown in Table 1. For molecular structure, VOCs include alkanes, alkenes, aromatic hydrocarbons, alcohols, aldehydes, ketones, and the like. In addition, polar and nonpolar VOCs are distinguished according to the degree of molecular polarity, and a detailed classification of common VOCs is shown in Figure 1.

VOCs can be divided into the following five categories: (a) Aliphatic hydrocarbons: aliphatic hydrocarbons are mainly derived from automobile exhaust, asphalt applications, biomass combustion, oil refining, agricultural products, and chemical processes. N-hexane is a common pollutant in the workplace and can cause harm to the human body after long-term exposure. (b) Aromatic hydrocarbons: aromatic compounds are usually produced from coal, automobile exhaust, oil refineries, and building materials. Toluene, benzene, and ethylbenzene are typical and are considered carcinogenic and hazardous due to their high toxicity and photochemical reactivity with ozone. (c) Halohydrocarbons: halogenated VOCs are mainly derived from industrial processes and water purification waste. Chlorinated volatile compounds with high volatility and strong persistence are the most common types that are widely present in environmental media. Persistent Cl-VOCs may have adverse effects on the environment and human health [3]. (d) Oxygen-containing organic compounds. VOCs include alcohols, aldehydes, ketones, ethers, phenols, esters, and acidic compounds and are mainly derived from building materials, industrial solvents, oil gases, and the coal chemical industry. Aldehydes are the most common VOCs. Formaldehyde is a major indoor pollutant, as it is widely used in building/furniture materials and consumer goods. Acetaldehyde is a by-product of ethanol combustion and a contributing factor to photochemical smog formation. Esters and ketones are mainly from furniture manufacturing, footwear industry, and printing industry; long-term exposure to esters and ketones can cause harm to the human body. (e) Compounds containing S and N. The compounds containing S and N can be mainly traced to the chemical oil combustion and leather industry. Aniline and Methanethiol are the two most common N/S-containing VOCs, respectively [4].

VOCs are a serious threat to the ecological environment and human health, and their treatment is urgently needed. Adsorption has become one of the most cost-effective methods for the treatment of VOCs due to its simple operation, high efficiency, and low energy consumption [5]. Traditional adsorption materials such as molecular sieves, activated carbon, and diatomite have the problems of small adsorption capacity, easy plugging, low selectivity, and difficult regeneration [6]. In view of the research on the adsorption and removal of VOCs by metal–organic framework (MOF)-based porous materials in the past 10 years, the classification of MOFs and the types of their composite materials were described in detail from the structure and characteristics of MOFs. Based on the influencing factors and mechanisms in the adsorption of VOCs by MOF-based porous materials, the research progress in the adsorption and application of VOCs by MOF-based porous materials was summarized, and the future development in this field was prospected.

## 2. Current Methods of Removing VOCs

### 2.1. Adsorption

**Figure 2:**
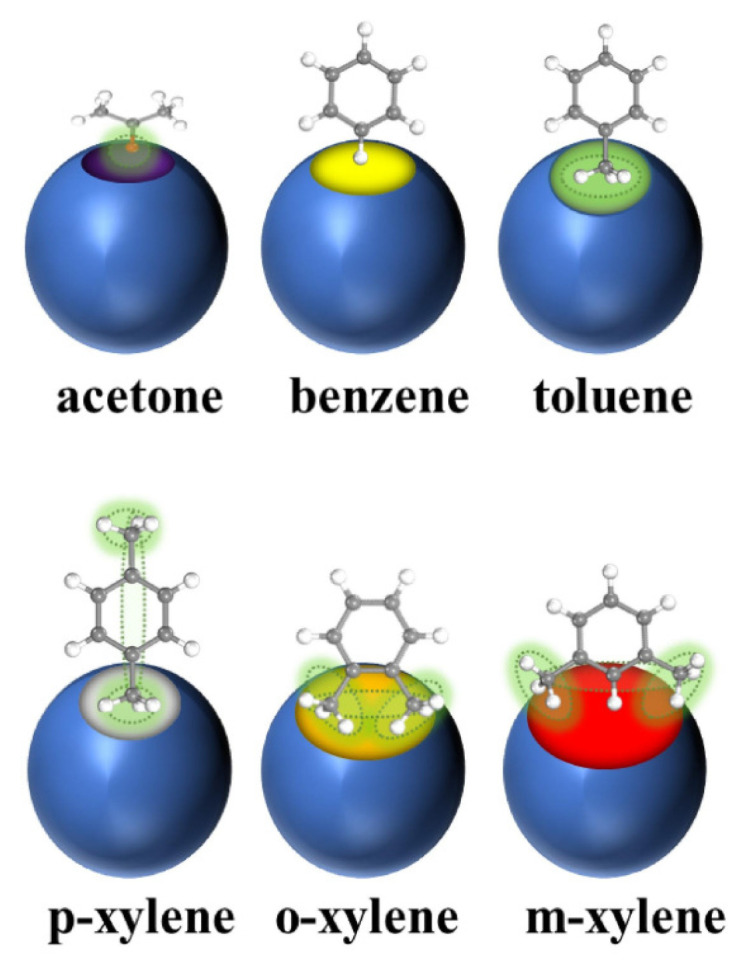
Scheme for entry of acetone, benzene, toluene, p-xylene, o-xylene, and m-xylene into UiO-67 pores [10].

The adsorption method refers to the method of separating VOC molecules from the atmosphere by using Van der Waals interactions or chemical bond interactions generated by the surface of the adsorbent and VOC molecules according to the adsorption selectivity of the adsorbent [7]. According to its adsorption type, it is divided into physical adsorption and chemical adsorption [8]. Compared with other methods, the adsorption method has the advantages of high removal efficiency, low energy consumption, low cost, mature process, etc. Although its treatment efficiency is affected by many factors, it is still one of the most widely used methods for the treatment of VOCs. Adsorption is considered one of the most promising VOC treatment technologies due to its high cost-effectiveness, flexible operation, and low energy consumption. There are many porous materials having a large surface area and a rigid structure that have been explored as adsorbents for the physical adsorption of toxic compounds. These materials include activated carbon, porous silicon dioxide, aluminosilicate zeolites, carbon nanotubes, resins, molecular sieves, and columnar clays [9]. Zhao et al. [10] performed experiments on the effect of the regulator ratio and guest molecule diffusion on the adsorption of VOCs by defective UiO-67 and showed a schematic representation of molecules entering the UiO-67 channel, as shown in Figure 2.

Factors that affect the efficiency of the adsorption method mainly include the characteristics of the adsorbent; the type, concentration, and nature of the adsorbed substances; and the temperature, pressure, and flow rate of the surrounding environment. As for the adsorbent, its specific surface area, porosity, and surface functional groups will affect its adsorption performance [11,12,13]. The larger the specific surface area, the smaller the pore size, and the larger the pore capacity of the adsorbent are conducive to the adsorption of VOCs and other pollutants. In addition, the design of appropriate functional groups on the surface of the adsorbent can also increase its adsorption performance, such as modification and doping [14,15,16,17,18,19]. At present, the most widely used and promising material is carbon material, which has the natural advantages of large specific surface area, abundant pore structure, large adsorption capacity, and other adsorbents, as well as the potential catalytic and surface chemical activity [11,20].

**Figure 3:**
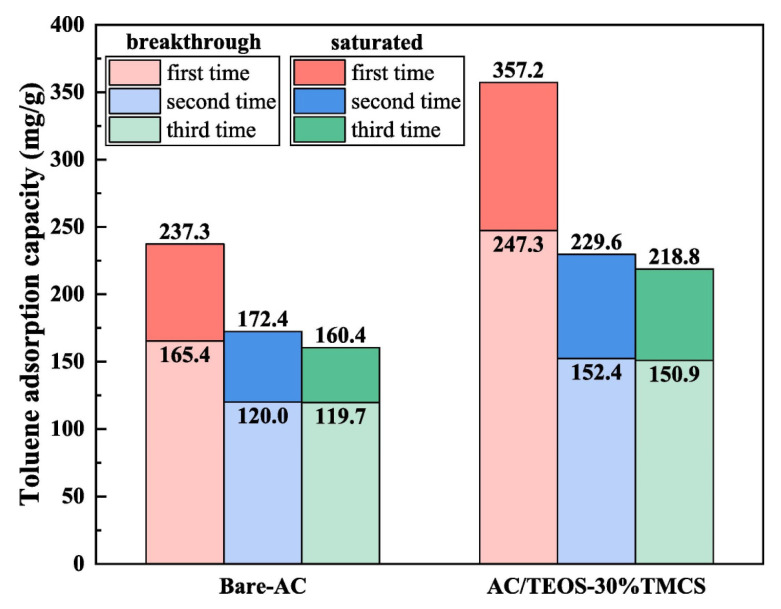
Breakthrough adsorption capacity and saturated adsorption capacity of toluene for regenerated ACs [21].

In order to develop effective adsorbents for certain VOCs, modifications or functionalizations are typically applied to improve the specific properties of the base adsorbent. In general, modifications to physical and chemical properties may be involved. For carbon materials, the surface chemistry and texture properties can be adjusted by acid, alkali treatment, impregnation with foreign functional groups, and heat treatment. Li et al. [21] investigated the adsorption of VOCs on trimethylchlorosilane-modified activated carbon under high humidity conditions and found that the TMCS-modified activated carbon maintained good VOC adsorption capacity under humid conditions, where the breakthrough and saturation adsorption capacities of the regenerated activated carbon for toluene are shown in Figure 3.

For the adsorption environment, the adsorption process is generally an exothermic process, so the low temperature is conducive to the adsorption. For example, Tsai et al. [22] found that within a certain temperature range, low temperature was more conducive to the adsorption of VOCs. In addition, excessive temperature may cause severe thermal movement of organic gas molecules to hinder adsorption. Moreover, the boiling point, molecular structure, and properties of the adsorbate will also affect the adsorption. For example, Zhu et al. [23] found that porous dopamine had a high selectivity for xylene under wet conditions. For VOCs, due to the variety and complexity, different adsorbents and adsorption environments will affect the adsorption effect.

VOCs in industrial organic exhaust gases are formed by the mixing of gases, and due to the different affinity of the components, competitive adsorption may occur in the mixed gas system. When the concentration of VOC vapor with strong adsorption affinity reaches a certain extent, competitive binding will inevitably form at the adsorption sites to replace VOC vapor with weak adsorption affinity [24]. Meng et al. [25] proposed the competitive adsorption process and related adsorption mechanisms between toluene, methanol, and acetone on ACF. In multi-component adsorption, the methanol and acetone were physically adsorbed mainly through dipole–dipole interactions. The adsorption of toluene is controlled by physical and chemical processes through the strong affinity between the adsorbate and the adsorbent. Since the aromatic ring of toluene acts as an electron receptor and carbonyl or lactone acts as an electron donor, a stable electron donor–receptor complex is formed. Notably, the stronger adsorption of toluene or acetone may replace the weaker adsorption of methanol. In addition, Vizhemehr et al. [26] found that the adsorption rate of the lighter compounds (MEK) was higher than that of the heavier compounds (n-hexane and toluene), and then the heavier compounds could replace the adsorbed lighter compounds, resulting in their forced desorption. Wang et al. [27] evaluated the eight VOC adsorption capacities of beaded AC and found the breakthrough curve of low boiling VOCs (n-butanol, n-butyl acetate) was rolled up. Their concentrations first increased above their influent concentrations, then decreased, and finally approached their influent values. This indicates that the low boiling compound is desorbed when replaced by a high boiling compound (2,2-dimethyl-propylbenzene), having a stronger adsorbate–adsorbent interaction.

### 2.2. Photocatalytic Degradation

Photocatalytic oxidation technology is considered to be one of the most promising pollution control technologies due to its strong oxidation capacity, small selectivity, mild conditions, and no secondary pollution [28]. Fujishima and Honda [29] first discovered in 1972 that TiO_2_ could decompose water into H_2_ and O_2_ under the action of photo-electricity, and thus, the photocatalytic technology was born. The application of photocatalysis in the environmental field began in 1976 when Carey et al. [30] applied TiO_2_ to the degradation of polychlorinated biphenyls (PCBs), a toxic organic substance in water. The basic principle is that when the photon energy is greater than or equal to the electron, the electron will be stimulated to transition and form a photo-generated electron–hole pair, which will continuously undergo oxidation–reduction reactions with the substances adsorbed on the surface of the catalyst under the illumination, thus degrading the pollutants or converting the light energy into chemical energy.

As a class of three-dimensional crystalline micro-/mesoporous hybrid material constructed from metal nodes interconnected with multi-dentated organic linkers, the modular structure of MOFs enables them to be facilely immobilized with photoactive sites for photocatalysis. Due to their high light collection capacity, adjustable pore structure, ordered pore structure, adsorption performance, high adsorption capacity, and narrow band gap or activation in the visible light range, MOFs have played a vital role in photocatalysis. Ramezanalizadeh et al. [31] successfully synthesized a novel, efficient, and recyclable MOFs/CuWO_4_ hetero-structured photocatalyst via a simple synthetic route and effectively photocatalytic degraded methylene blue and p-nitrophenol organic pollutants under mild reaction conditions, with the maximum removal rates of methylene blue and p-nitrophenol reaching 98% and 81%, respectively. In addition, MOFs/CuWO_4_ hetero-structured photocatalyst has good reusability and can be recycled six times, with the degradation rate of the two organic substances still reaching about 80%. However, the removal rates of methylene blue and p-nitrophenol by CuWO_4_ alone are only about 40% and 20%. The one-step hydrothermal synthesis of MOFs/reduced graphite oxide (GO) by Thi et al. [32] has a photocatalytic degradation efficiency of more than 90% for a variety of organic dyes such as methylene blue, methylene orange, and rhodamine B under 20 min sunlight, which is much higher than the degradation rate of organic pollutants when the reduced GO is used alone. The synergistic effect generated by the hybrid combination of MOFs/reduced graphene oxide plays a vital role in delaying the photogenerated electron–hole recombination rate and maximizing the charge transfer of the whole system, thereby realizing the high-efficiency photocatalytic performance.

However, at present, most of the photocatalysts have the light absorption range in the ultraviolet region, photo-generated-hole pairs are easily recombined, and the active sites adsorbed on the catalyst surface are few. These defects limit the practical application of this technology. Therefore, in order to improve the activity of photocatalysts, many researchers have modified them by various methods. At present, the modification methods of photocatalysts mainly include element doping, surface modification, semiconductor recombination, morphology regulation, etc. Among them, element doping can not only change the lattice parameters of the photocatalyst (such as introducing defects or vacancies) to effectively separate photo-generated electron–hole pairs, but also adjust the band–edge position and band gap of the energy band of the catalyst, thereby improving the quantum efficiency of the photocatalytic material [33,34,35,36,37,38,39,40]. In 2001, Asahi et al. first found that after some O atoms in TiO_2_ were replaced by N atoms, the doping of N atoms could reduce the valence band potential of TiO_2_ and the band gap width of the semiconductor, thus improving the light absorption capacity of TiO_2_ [40]. In addition, metal ion doping can also modify the catalytic activity of the photocatalyst. For example, Choi et al. found that doping transition metal ions into TiO_2_ could significantly improve its photocatalytic ability [39]. By means of the appropriate substitution of common aromatic linkers or by selecting dyes as linkers, it is possible to design MOFs with a visible-light photoresponse at long wavelengths. This methodology to control the light-absorption properties is typical of organic molecules, and these concepts can be easily implemented in MOFs, but are not suitable for conventional inorganic semiconductors [41].

## 3. MOFs as Adsorption and Degradation Materials

### 3.1. Overview of MOFs

In the past half-century, grid chemistry has developed into a powerful auxiliary tool for the design and synthesis of porous crystalline skeletal materials. As an important subset of coordination grid chemistry, MOFs are kinds of materials consisting of a supramolecular microporous network structure formed by the coordination and self-assembly of organic ligands and metal ions, which were first discovered by Hoskins and Robson [42]. Compared with other traditional adsorption materials, MOFs have the characteristics of large specific surface area, abundant pore structure and easy adjustment, diverse structural compositions, strong regeneration, open metal active centers, and chemical modifiability. The research has focused on gas adsorption, separation and storage, catalysis, sensing, etc., among which MOFs showed very good application prospects in the adsorption and removal of VOCs [43].

MOFs are attractive organic–inorganic porous materials consisting of inorganic secondary structural units (metal oxide clusters or metal ions) coordinated with organic moieties [44]. Such coordination polymers have significant chemical and structural diversity and are superior to conventional porous materials in the adsorption of harmful gases/vapors due to the ability to fine-tune the pore size and chemistry of the MOFs at the molecular level [45]. Depending on the size and chemical characteristics of the adsorbate, MOFs have the potential to design and manufacture well-defined adsorption sites immobilized on ligands and certain pore sizes by altering the nature of the linker or organic linker, with great potential for gas/vapor adsorption applications. In general, the quantum size nodes of MOFs connected by transition metal ions to the oxygen of the organic linker provide a large number of open metal sites devoid of electrons, which facilitates the chemisorption of the gas/vapor by adsorbate–surface interactions. In addition, MOFs are highly crystalline solid materials with semiconducting properties that exhibit significant photocatalytic activity for the degradation of organic contaminants with comparable thermal stability. Thus, these unique properties give MOFs a “self-cleaning” capability in which the adsorbed gas/vapor degrades in situ under light irradiation. Therefore, the purification of gaseous pollutants from the environment by MOFs may play a vital role in the near future [9].

Different kinds of porous MOFs are composed of metal ions or clusters of metal ions coordinated to organic ligands in an ordered one-, two-, or three-dimensional framework [46]. Over the past two decades, MOFs have attracted worldwide attention due to their excellent properties, such as ultra-high and specific surface area (up to 3000 m^2^ g^−1^), excellent thermal stability (>400 ℃), customizable pore structure, and easy functionalization [47]. The vast potential of MOFs for gas storage, separation, heterogeneous catalysis, and sensing has been well explored [48]. The open metal sites on the pore surface of MOFs can be used to enhance the adsorption of various VOCs. Unlike conventional adsorbents, MOFs are capable of maintaining their permanent structure and crystallization order after regeneration [49]. As an emerging class of porous materials, MOFs have the characteristics of high pore volume and specific surface area. In addition, they have an open and ordered pore structure, rich interconnected 3D channels, ultra-high porosity, and customizable chemical properties [50].

### 3.2. Structural Classification and Properties of MOFs

#### 3.2.1. Classification of MOFs

MOF materials have been continuously studied and synthesized with an increasing number of types. According to the components and preparation methods, they can be divided into: isoreticular metal–organic frameworks (IRMOFs), porous coordination networks (PCNs), zeolitic imidazole frameworks (ZIFs), materials of institute lavassier frameworks (MILs), etc. The structures and specific compositions of each type of MOF described below include the selection ranges of organic ligands or metal ions.

(1) IRMOF material

**Figure 4:**
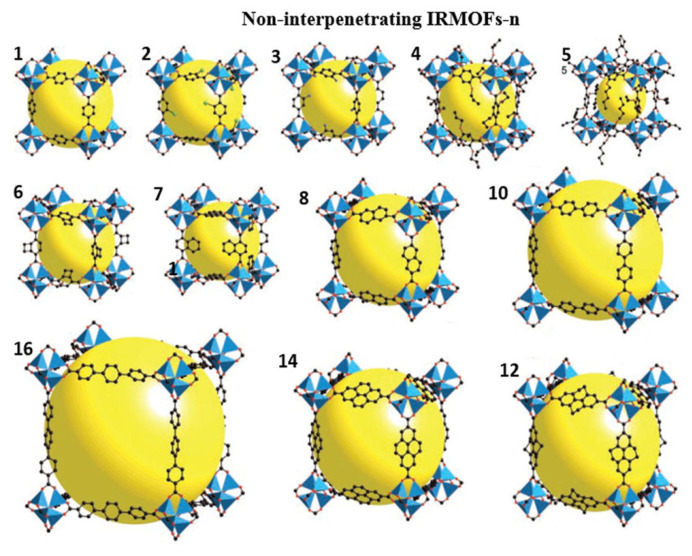
Structures of non-interpenetrating IRMOF-n (*n* = 1–8, 10, 12, 14, and 16) [52].

This material is the most representative kind of MOF material. Among them, IRMOF-1, or MOF-5, has been studied the most. It was first designed and synthesized by Yaghi’s research group [51] with the help of the idea of metal carboxylate cluster chemistry, i.e., a three-dimensional metal–organic framework material with a simple cubic structure constructed with rigid organic ligand terephthalic acid (BDC) and transition metal Zn by the solvothermal method. The MOF-5 had excellent adsorption performance for VOCs such as benzene (802 mg/g), dichloromethane (1211 mg/g), and trichloromethane (1367 mg/g) at 22 °C, which was 4–10 times that of traditional adsorption materials such as zeolite and activated carbon. It was further found that the adsorption capacity of IRMOF-3, MOF-74, and MOF-199 for VOCs such as benzene, dichloromethane, and ethylene oxide was at least 59 times higher than that of pitch-based activated carbon. The regular branched structure of IRMOFs makes it possess the characteristics of large specific surface area and high porosity on the basis of three-dimensional structure, making it widely used in adsorption, separation, storage catalysis, and other fields. Synthesis and application of plasma metal–organic frameworks IRMOFs-n (*n* = 1, 3, 6, 8) were carried out by Eddaoudi et al. [52]. Figure 4 is an illustration of IRMOFs-n (*n* = 1–8, 10, 12, 14, 16), which are non-interpenetrating IRMOFs.

(2) PCN material

**Figure 5:**
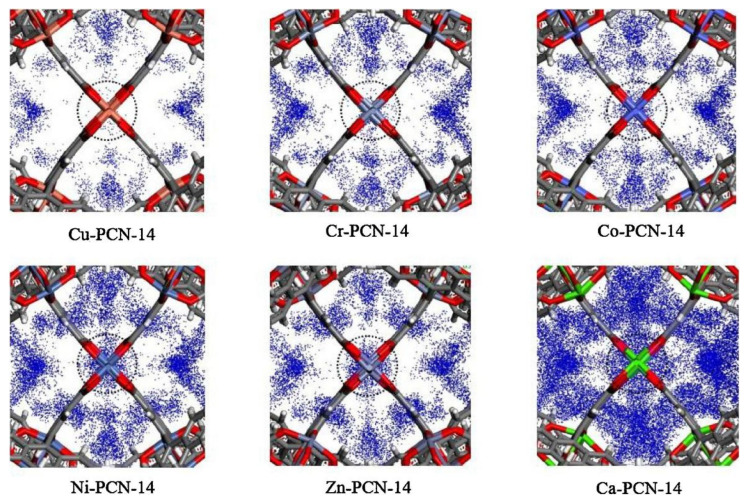
14CH_4_ density at the OMS of M-PCN-14 at 5 Bar of methane [54].

This material is a porous material formed by multiple octahedral cubic solid pore cages. Since 2006, Zhou’s Research Group [53] has successively synthesized PCN-9 and PCN-14 using a bionic method of coordinating unsaturated metal centers (UMCs) and endogenous metal centers (EMCs). The framework structure of this material contains a large number of benzene ring structures, and the pore size is conducive to methane diffusion. Studies have shown that the presence of EMCs can significantly enhance the affinity of materials for specific gases. Among them, PCN-14 has a specific surface area of 2176 m^2^/g and a pore volume of 0.87 cm^3^/g. Under the conditions of 3.5 MPa pressure and temperature of 290 K, it has excellent storage performance for methane (230 *v*/*v*), which exceeds 28% of the required standard (180 *v*/*v*) of the United States Department of Energy. The effect of metal substitution on 14CH_4_ storage in the PCN-14 metal–organic framework was computationally assessed by Sun et al. [54]. To investigate the effect of different metals on absorption, they modeled the 14CH_4_ density at the open metal sites (OMS) of M-PCN-14 at 5 Bar of methane, as shown in Figure 5. Cu had low methane adsorption, and the 14CH_4_ density at the OMS increased with metal substitution, corresponding to the increase in methane adsorption. In particular, Ca had a very strong interaction with 14CH_4_, such that the volumetric adsorption of Ca-PCN-14 was one of the highest in the M-PCN-14 series despite the low density of its framework.

(3) ZIF material

Yaghi’s research group [55] first proposed ZIF material, which is a tetrahedral framework material formed mainly by the reaction of imidazole ligand (Ims) with Co(Ⅱ) or Zn(Ⅱ) to synthesize imidazole-coordinated zeolite-like complexes [56]. Then, continuously being researched and developed, a series of ZIF materials with different pore channel structures and surface chemical properties are synthesized by changing functional groups or organic ligands on the premise of not changing the topological structure, and the ZIF materials have increasingly abundant structures, functions, and the like, and are simple and easy to operate in synthesis processes, good in chemical stability, low in cost, and easy to obtain in raw materials [57]. Among the many ZIFs synthesized, some of them have exceptional thermal and chemical stability [55], and exhibit great promise for gas storage and separation [58].

**Figure 6:**
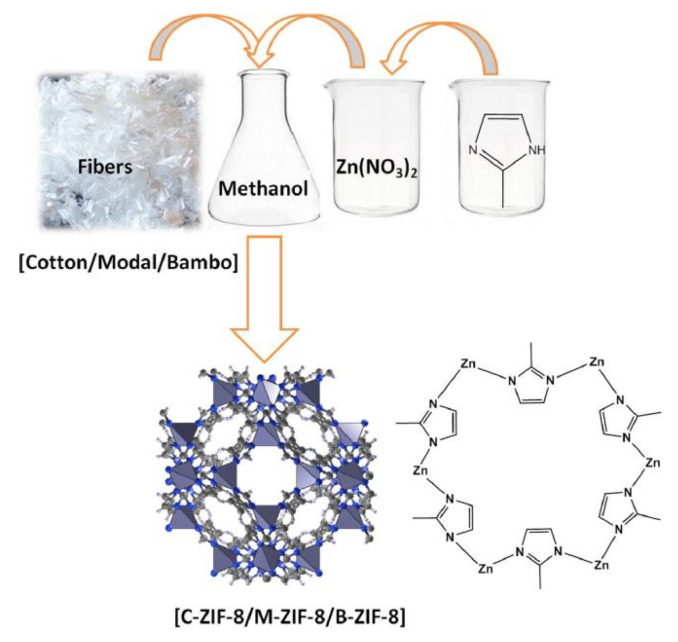
Synthesize scheme of ZIF-8 using different cellulosic fiber templates [63].

Many studies have been carried out on the adsorption and diffusion of pure gases in ZIFs. Wu et al. [59] investigated the H_2_ adsorption point and binding energy in ZIF-8 using a combination of experimental and computational methods. Among the available theoretical studies, Zhong and coworkers investigated the sorption and diffusion properties of CO_2_ in two typical ZIFs (ZIF-68 and ZIF-69) [60] and also studied the effect of the framework charge on CO_2_ uptake in some ZIFs [61]. Xie et al. [62] prepared amino-functionalized ZIF-8 by hydrothermal synthesis. The study found that there were a large number of microporous structures, abundant open metal sites, and functional groups in ZIF-8, which exhibited outstanding adsorption performance for VOCs, with an adsorption capacity of 89.95 mg/g for formaldehyde. Emam and Abdelhameed [63] designed a scheme for the synthesis of ZIF-8 using different cellulose fiber templates (Figure 6) in order to study the separation of anthocyanins from Lahia extract using a cationic nanoelectrode ZIF-8 constructed from a detachable template.

(4) MIL material

**Figure 7:**
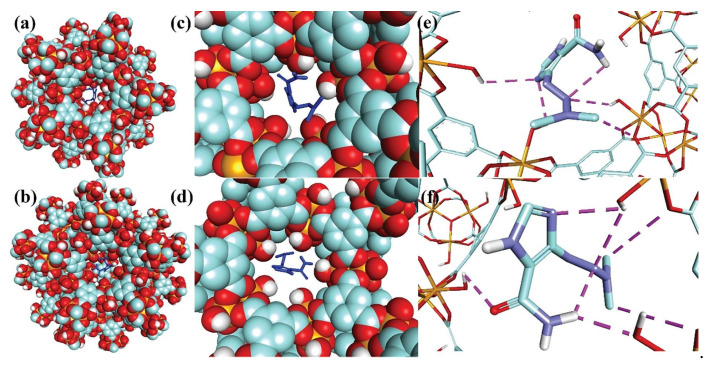
Schematic representation of the molecular docking of MIL-100(Fe) small (**a**,**c**,**e**) and big (**b**,**d**,**f**) cages with DTIC. (**a**,**b**) Full view from outside. (**c**,**d**) View from inside the cage. (**d**,**f**) Hydrogen bonds between DTIC and pentagonal window. Fe, C, O, and H colors are orange, green, red, and white in cage structures, respectively. The purple line in (**c**) indicates hydrogen bonding [66].

It was first combined by Ferey from the University of Versailles in France [64]. According to the synthetic methods, it can be divided into two types: one is synthesized by trivalent metal ions (such as iron, aluminum, chromium, or vanadium) with organic ligands such as terephthalic acid, and the other is synthesized by lanthanide and transition metal elements with dicarboxylic acids such as glutaric acid. MIL materials have the advantages of strong flexibility, high water stability, and good selectivity. Among them, MIL-53, MIL-100, and MIL-101 are the most representative and have attracted extensive attention. They are often used in the fields of membrane separation, heterogeneous catalysis, and gas storage [65]. Barjasteh et al. [66] used rapid and green synthesis techniques to synthesize MIL-100 (Fe) MOFs. The synthesized MOF particles had a high adsorption capacity of 292.87 mg/g for dacarbazine (DTIC) under optimal conditions, and the schematic diagram of the molecular docking between MIL-100(Fe) size cages and DTIC is shown in Figure 7.

(5) Other structures

In addition, the synthesis of some MOF materials with other structures such as UiO and HKUST-1 also made this family larger [67]. Based on UiO-66, modified UiO-66 was synthesized by the solvothermal method by Zhang et al. [68] using polyvinylpyrrolidone (PVP) as a structure-directing agent to regulate its morphology and pore structure. The research showed that the addition of PVP affected the self-assembly of organic ligands and metals, causing C=O to rupture and then forming modified UiO-66 with ligand defect sites. Because the ligand defect site in the modified material was the main active site for toluene adsorption, therefore, modified UiO-66 had a higher toluene adsorption capacity than UiO-66. In recent years, as a copper-based MOF material, HKUST-1 has been widely used for the adsorption and separation of gases. Tian et al. [69] synthesized and found that HKUST-1 was an adsorbent with high selective adsorption and separation performance for dichloromethane and trichloromethane. At 298 K, the adsorption capacity of HKUST-1 for the two was up to 13.12 mmol/g and 8.53 mmol/g, respectively, which was much higher than that of other adsorbents such as activated carbon and zeolite under the same conditions.

#### 3.2.2. Classification of MOF Composites

Although MOF materials have many advantages, they still have some shortcomings, such as low mechanical strength, solid powder, poor stability of water, and so on. In order to improve these shortcomings and make them meet the needs of practical applications, a series of MOF composites have appeared one after another. MOF composite material is a binary or multi-component composite material synthesized by taking MOFs as a matrix or a reinforcing body and taking other substances with specific functions as the reinforcing body or the matrix, and different components in the composite material can effectively introduce multifunctionality and synergistic effects so that the original performance of the MOF material is improved, the properties and functions of the MOF material are expanded, and the MOF composite material has good application in the aspect of absorbing VOCs [43]. At present, the main materials that can be combined with MOFs are metals, molecular sieves, carbon-based porous materials, magnetic nanoparticles, polymers, and quantum dots. In this paper, several common types of MOF composites are introduced according to their different composition types from MOFs.

(1) MOF-Metal composite

At present, transition metal elements are mostly used as metal elements for synthesizing MOF composite materials. Transition metal ions easily form multi-core metal clusters with stable thermodynamics and clear structure when forming MOFs, and the final framework structure is easy to predict and strong in designability, such as Co, Mn, Ni, and the like, and has the characteristics of low cost, green environment protection, good absorbability, etc. Qin et al. [70] prepared Co-M-MOFs (M=Cu, Mn, Ni, and Zn) by an ion-assisted solvothermal method. Cu^2+^, Mn^2+^, Ni^2+,^ and Zn^2+^ could be well-doped into the Co_3_O_4_ lattice, and there were a large number of adsorbed oxygen and oxygen vacancies on the surface of the material, which realized the high performance and stability of the composite. The synthesis of [Ga_2_(OH)_2_(L)] (H_4_L = biphenyl-3,3′,5,5′-tetracarboxylic acid), designated MFM-300(Ga_2_), (MFM = Manchester Framework Material replacing NOTT designation), by the solvothermal reaction of Ga(NO_3_)_3_ and H_4_L in a mixture of DMF, THF, and water containing HCl for 3 days was reported by Krap et al. [71]. The Fe(III)-based material [Fe_3_O_1.5_(OH)(HL)(L)_0.5_(H_2_O)_3.5_], MFM-310(Fe) was also synthesized by a hydrothermal method using Fe(NO_3_)_3_ and the same ligand as raw material. It was shown that the Fe-doped MFM-300(Ga_2_) had a positive effect on the gas adsorption capacity, especially for CO_2_, where the CO_2_ adsorption capacity of MFM-300 (Ga_1.87_Fe_0.13_) was increased by 49% compared to the same metal base material. Secondly, Ca, Mg, Al, and other metal elements have a smaller radius and higher charge, strong polarization ability, and more covalent bonds with the coordination bonds formed by oxygen-containing ligands. Therefore, the formed MOF composite has high chemical and thermal stability. Rare earth metal ions have high coordination number and flexible coordination mode. The coordination of unsaturated metal ions existing in an MOF structure can be used as high-performance active sites. Therefore, the introduction of rare earth metal ions can achieve the directional synthesis of MOF–metal composites. In addition, it has been found that due to the high porosity and large number of microporous structures of MOFs, noble metal nanoparticles such as Pd and Pt can be successfully encapsulated into the pores, which limits the growth of metal particles and ensures their good dispersibility.

(2) MOF–Carbon-based composite

**Figure 8:**
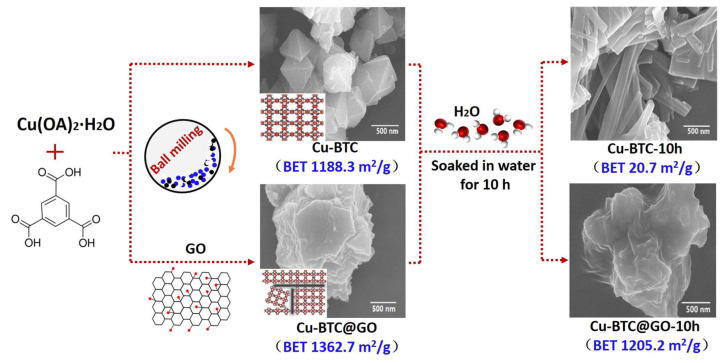
Synthesis pathways for Cu-BTC-10h and Cu-BTC@GO-10h [74].

Carbon-based porous materials, including activated carbon (AC), GO, and carbon nanotubes (CNT), have excellent water resistance and structural stability. The combination of that carbon-based porous material with MOFs can not only effectively enhance the stability of the MOFs, but also provide excellent diffusion channels for gas molecules due to the abundant mesopores and macropores in the carbon-based porous material, thereby remarkably improving the gas adsorption capacity of the material. Li et al. [72] prepared AC/Cu-BTC composites by the hydrothermal method to study the adsorption performance of alkane VOCs (n-hexane) before and after water absorption. AC provided the interaction substrate for the nucleation and growth of Cu-BTC, and then affected its specific surface area parameters, resulting in the reduction of the specific surface area of AC/Cu-BTC, thus reducing its adsorption capacity. Sun et al. [73] studied the adsorption capacity of GO/Cu-BTC and RGO/Cu-BTC for different adsorbates (methane, ethane, ethylene, and propylene). The adsorption capacity of the composites for gas molecules was related to the surface properties of the graphene samples. As the surface functional groups of the materials had electrostatic repulsion and hydrophilicity, they promoted their uniform dispersion and inhibited their agglomeration behavior in the preparation process, thus increasing the specific surface area. In addition, by controlling the reduction degree of RGOs, selective adsorption of gas by the material can be achieved. Li et al. [74] synthesized Cu-BTC@GO and verified its adsorption performance for toluene. The synthesis pathways for Cu-BTC-10h and Cu-BTC@GO-10h are shown in Figure 8. The toluene adsorption capacity of Cu-BTC@GO-5 was much higher than that of traditional activated carbon and zeolite. This is due to the increase of the specific surface area of Cu-BTC@GO-5 on the one hand, and the increase of the surface dispersibility of Cu-BTC due to the introduction of GO.

(3) MOF–Molecular sieve composite

The organic combination of MOFs and a molecular sieve can effectively solve the problem of low selective separation of a traditional molecular sieve. Li et al. [75] adopted the hydrothermal method to synthesize Cu-BTC, ZSM-5, and Cu-BTC@ZSM-5 and studied the dynamic adsorption process of toluene on different adsorbents. It was found that the high specific surface area and hydrophobicity of Cu-BTC@ZSM-5 could improve the competitive adsorption of toluene to water vapor. Furthermore, the addition of ZSM-5 provided more active sites for adsorption. Saini et al. [76] synthesized an MOF–zeolite composite foam (ZMF) to study its selective adsorption capacity for three common indoor VOCs (benzene, n-hexane, and cyclohexane). The selectivity of ZMF to VOCs is significantly higher than that of MOF-199 and zeolite foams (ZF) because VOC molecules easily enter the surface of MOF-199 in the zeolite structure to improve the adsorption performance of ZMF. In addition, the introduction of MOF-199 altered the physical and surface properties of the zeolite foam to improve the material properties.

(4) MOF–Magnetic nanoparticle composite

The magnetic substances (such as Fe_3_O_4_, Fe_2_O_3_ and other iron oxides) and the MOF material are combined to form the MOF–magnetic nanoparticle composite material, and under the action of an external magnetic field, the excellent magnetism of the MOF–magnetic nanoparticle composite material is utilized to effectively separate and improve the renewable capacity of the material. Additionally, the MOF–magnetic nanoparticle composite material has the advantages of high specific surface area, larger pore diameter, superparamagnetic, high selectivity, good dispersibility, reusability, and the like, and is widely applied to gas adsorption, harmful substance removal, drug delivery, and the like. The combination of the MOF metal cations and ligands results in variable geometries and electronic structures that can be tuned over a wide range of pore sizes and specific surface areas. The unsaturated metal ion center generated by the combination of the unsaturated metal site and the organic ligand has the effect of absorbing and separating gas through the coordination effect with the VOC molecule. MOFs used for the adsorption of VOCs mainly include the IRMOF series and MIL series [77]. Adenine-based MOFs, often referred to as bio-MOFs, have applications in water treatment [78], drug delivery [79], fluorescence sensors [80], CO_2_ capture [81], and gas separation [82]. Adenine is a multifunctional biomolecule. In addition, the unique coordination pattern of bio-MOF-11 contributes to the generation of more free Lewis base sites than other bio-MOFs36-38s, and thus, has considerable potential for the removal of VOCs [83].

### 3.3. Synthesis Methods and Categories

MOF can be synthesized by using the colloidal deposition method, rapid mechano-chemical method, hydrothermal method, solvothermal method, ultrasonic synthesis method, microwave synthesis method, evaporative solvent method, diffusion method, etc. [2].

#### 3.3.1. Colloidal Deposition Method

Considering the unprecedented VOC adsorption capacity of MOFs and the excellent photothermal effect of Ag nanoparticles, Liu et al. [50] adopted the colloidal deposition method to combine Ag nanoparticles with UiO-66 and constructed a novel photodynamic Ag/UiO-66 adsorbent. The obtained Ag/UiO-66 samples were found to have excellent porosity and high styrene adsorption capacity of the parent UiO-66.

#### 3.3.2. Rapid Mechano-Chemical Method

Li et al. [84] reported the preparation of Cu-BTC for the adsorption of benzene by rapid mechanochemistry. Cu-BTC has a larger specific surface area than that prepared by the hydrothermal method, reaching 1442.7 m^2^ g^−1^; the adsorption capacity of benzene is 6.90 mmol g^−1^ at 298 K and 8 kPa. Compared with traditional adsorbents, the adsorption capacity of Cu-BTC in this work was 1.38 times and 2.2 times that of activated carbon [85] and zeolite NaY [86], respectively.

#### 3.3.3. Hydrothermal Method

The hydrothermal method prepares raw materials and water into a solution according to a certain proportion, and then organic ligands are added, and simultaneously an ethanol solution can be added, and the solution is put into a reaction kettle and heated in a closed manner to react under self-pressure. Under normal circumstances, the temperature is 80–200 °C. Another organometallic framework material, MFM-520, was reported by Yang’s research group [87] and synthesized by Lin’s research group [88]. Soaking saturated NO_2_@MFM-520 in water for 10 min and stirring to obtain nitric acid solution for reuse as a chemical raw material. The specific surface area of MFM-520 remained unchanged after 125 cycles of NO_2_ adsorption, and there was no loss of adsorption capacity.

#### 3.3.4. Solvothermal Method

**Figure 9:**
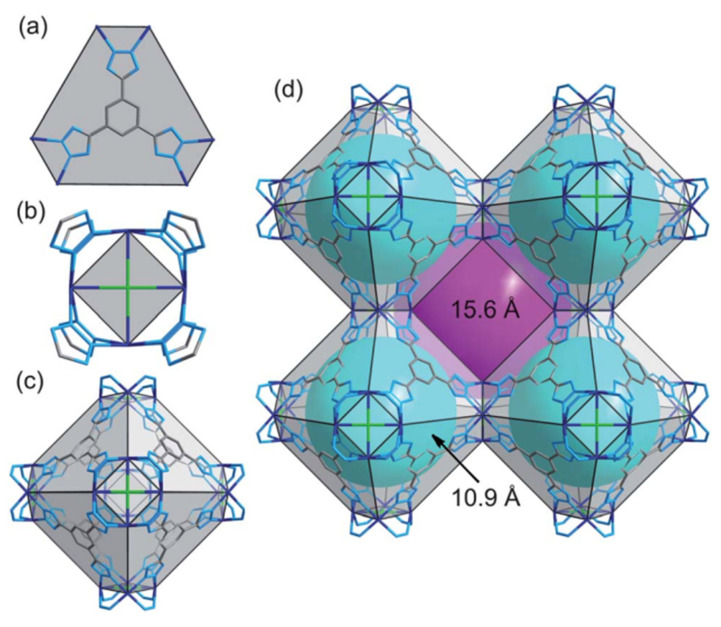
Views of the frameworks of 1 and 2 in wire representation (color codes: N, light blue; Co or Cd, dark blue; C, grey, Cl, green). (**a**) BTT ligand connected with six neighboring M^2+^ ions constructing the 3-connected node and hexagonal face. (**b**) Four M^2+^ ions bridged by eight tetrazolate rings forming the 8-connected node and square face. (**c**) A sodalite-like cage constructed from six [M_4_Cl]^7+^ units and eight BTT linkers located at its square and hexagonal faces, respectively. (**d**) A cube of eight cages, each of which is connected with six adjacent cages through its square faces. The void cavity inside the cages and the one formed by connecting eight such cages are displayed as cyan and magenta spheres, respectively. For clarity, hydrogen atoms and coordinated water molecules have been removed from all structural plots [95].

The solvothermal method and hydrothermal method are basically the same and have the same principle. The only difference is that the solvent of the solvothermal method is no longer water, and it can also be an organic solvent with different functional groups. N,N-dimethylformamide (DMF) and N,N-dimethylformamide (DEF) are often used. Most of these solvents are slightly toxic and pollute the environment. Using zirconium tetrachloride (ZrCl_4_) and terephthalic acid (BDC) as raw materials, Cavka et al. [89] synthesized UiO-66 by the solvothermal method in organic solvent DMF. The BDC ligand was changed into 4,4-biphenyldicarboxylic acid (BPDC) with large pore size, and UiO-67 was obtained by the same method. Ebrahim et al. [90] found the structural characteristics and stability of zirconium-based MOFs, indicating that they are suitable for use as adsorbents for NOx. Later, Ebrahim et al. [91] modified Zr-MOF, synthesized by the solvothermal method with the introduction of -NH_2_ of melamine and urea, and found that the adsorption of NO_2_ by urea-modified Zr-MOF was increased under wet conditions, which was related to the presence of water. In addition, they demonstrated that the incorporation of metal active sites could provide more adsorption sites. Therefore, they modified Zr-MOF with Ce^3+^ and synthesized Ce-UiO-66 and Ce-UiO-67 according to the previous method [90] to study their adsorption efficiency for NO_2_ [92]. Compared with the previous result [90], the use of Ce doping not only significantly improved the porosity, but also enhanced the structural stability and resistance to NO_2_ corrosion. Caskey et al. [93] used 2,5-dihydroxybenzene -1,4-dicarboxylic acid and zinc nitrate tetrahydrate as raw materials, which were dissolved in DMF solvent, to synthesize porous material MOF-74 by the solvothermal method. Using zinc nitrate hexahydrate as the raw material, Tan’s research group [94] also adopted the solvothermal method to obtain Zn-MOF-74 and synthesized Mg-MOF-74, Ni-MOF-74 and Co-MOF-74 to study the adsorption characteristics of NO_2_. Two new microporous MOFs, Co-BTT(1) and Cd-BTT(2), were prepared by Biswas et al. [95] using 1,3,5-benzenedimethazole (H_3_BTT) as a linker in two different amide solvents (DMA for 1; DMF for 2) using a solvothermal method; the structures are shown in Figure 9. The successful synthesis and characterization of these two compounds promotes the feasibility of synthesizing microporous M-BTT frameworks containing mostly 3D (Mn, Fe, Co, Cu) and 4D (Cd) transition metal (II) ions. Both compounds are highly porous and have promising applications in gas phase adsorption and storage.

#### 3.3.5. Ultrasonic Synthesis Method

**Figure 10:**
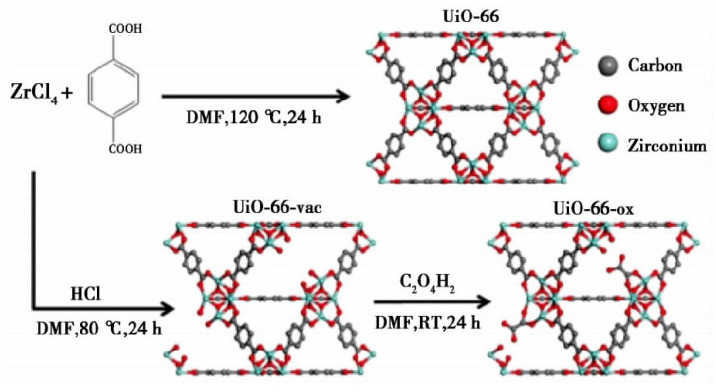
Synthesis of UIO-66, UiO-66-vac, and UiO-66-ox [101].

Ultrasonic synthesis, also known as chemical synthesis, utilizes ultrasonic waves to generate strong large shock wave energy based on solvothermal synthesis, thus shortening the reaction time and synthesizing particles with smaller particle size [96]. Compared with the solvothermal method, the ultrasonic synthesis method can obtain higher product yield in a short time, but has a higher cost. Xiao et al. [97] synthesized HKUST-1(MOF-199 and Cu-BTC), and then subjected it to ultrasonic treatment in a solution mixed with ethanol and deionized water at an equal ratio for 5–10 min after crystallization, indicating that HKUST-1 was a good gas storage material with environmental applicability. Levasseur et al. synthesized the precursor material HKUST-1 according to the method of Petit et al. [98] and GO according to the method of Seredych et al. [99]. Then, GO powder was added to the solvent solution and dispersed in HKUST-1, and sonicated for 5 min to ensure the complete dissolution of the crystals. It was found that under the drying conditions, the functional group of GO would interact with the copper site of MOF and form new pores in the interface between the carbon layer and the MOF, thus improving the adsorption efficiency. Under humid conditions, the adsorption efficiency decreased due to the competitive adsorption of water and NO_2_, and the NO decreased due to the disproportionation reaction of adsorbed NO_2_. Using the method developed by Katz et al. [100] and supplemented with ultrasonic treatment for 20 min, Decoste et al. [101] created a vacancy in the synthesized UiO-66 due to the lack of a linking group, which was called UiO-66-vac. Then, the oxalic acid solution was used to react with UiO-66-vac to obtain UiO-66-ox, and the general synthetic steps are shown in Figure 10.

#### 3.3.6. Microwave Synthesis Method

Microwave synthesis is a new method that has arisen in recent years. It is different from the solvent (water) thermal method in the heating method; the microwave is an electromagnetic wave. When it comes into contact with any substance, electromagnetic waves will undergo charge movement and direct contact, so the occurrence time is shorter and the heat transfer is faster. The solution is usually heated by microwave for 1 h or more to obtain crystals with nano-scale (usually 15 nm) particle size [102]. Peterson et al. [103] used ZrCl_4_ and 2-aminoterephthalic acid to dissolve in DMF solvent and synthesized pale-yellow powder UiO-66-NH_2_ by the microwave-assisted solvothermal method. The lack of significant change suggested that the material is essentially structurally intact with no skeletal bond cleavage upon exposure to NO_2_. They improved the adsorption capacity of NO_2_ and reduced the generation of by-product NO relative to UiO-66 [101].

#### 3.3.7. Evaporative Solvent Method

**Figure 11:**
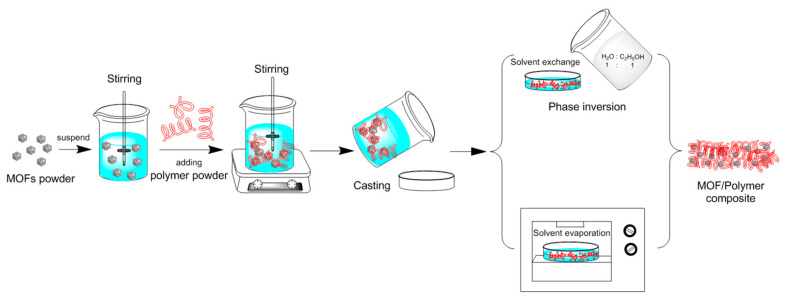
MOF/polymer composite prepared by evaporative solvent method [104].

The evaporation solvent method is a synthetic method in which a mixed solution of a metal salt and an organic ligand required for the preparation of MOF is saturated by direct evaporation at room temperature to precipitate the target product as a crystal. This method has a simple and rapid synthetic process, and the reaction can be completed within a few hours. In addition, the direction of the entire reaction can be adjusted by changing the amount of solvent added in the early stage of the reaction. Guo et al. [104] dispersed the MOF powder into a solvent by mechanical stirring, and then dissolved the polymer into the MOF solution to obtain an MOF/polymer suspension. The solvent in the suspension was then evaporated by solvent evaporation, followed by precipitation of polymer and MOF to obtain MOF/polymer composite. The whole synthesis process is shown in Figure 11. The evaporative solvent method is a common method for preparing MOF/polymer mixed matrix films. However, due to the incompatibility between MOF and polymer, MOF in suspension is agglomerated, and MOF in composite is not uniformly distributed step by step.

#### 3.3.8. Diffusion Method

**Figure 12:**
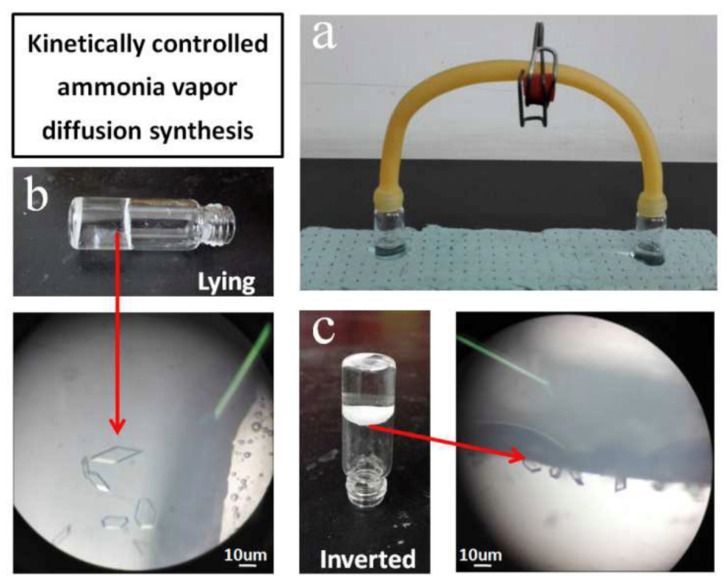
Controlled vapor diffusion synthesis of Zn(INA)_2_(H_2_O)_4_ at room temperature. (**a**) apparatus for the synthesis; the reaction mixture was in the left-hand vial, ammonia solution was in the right-hand vial. (**b**) the vial lying on its side, crystals on its side wall. (**c**) the vial inverted, crystals at the interface [105].

The diffusion method is a common synthesis method of MOF, which can be divided into gas phase diffusion method, liquid phase diffusion method, and gel diffusion method. In the gas phase diffusion method, the deprotonated agent was added into the reaction precursor solution to promote the ligand to react, and then further interacted with the metal ions in the system to structure the target MOF. The liquid phase diffusion method commonly used in that research requires that metal salts and organic ligand are dissolved in different solvents, and the two solutions react through liquid surface contact by a special device to generate the require metal–organic framework crystals. Yang’s research group [105] proposed a unique synthesis method of Zn(INA)_2_(H_2_O)_4_ based on the Zn(II) complex, namely, the synthesis was conducted at low temperature by kinetically controlling the vapor diffusion input of ammonia (NH_3_) in the absence of external energy (Figure 12). The synthetic conditions required by this method are mild, and the generation process of MOFs can be observed. However, the prerequisite for using this method is that the reactants have good solubility at room temperature. In the gas phase diffusion method, solutions of ligands and catalysts need to be prepared, and then the two are connected to stand at normal temperature and pressure for a long time to form metal organic framework materials through molecular diffusion and other processes. However, this method requires catalysts with good volatility. The single crystal suitable for X-ray diffraction analysis can be obtained by the diffusion method, which is convenient for analyzing the material structure with the aid of an X-ray diffraction analyzer. The disadvantage is that that reaction requires a long time and the yield is very low, so it can only be used for research on the crystal structure.

## 4. Mechanism and Application of MOFs for VOC Removal

### 4.1. Types and Influencing Facters

It is well known that the micropores of the adsorbent, especially the narrow micropores, play a key role in the adsorption of VOCs, and there is a clear correlation between adsorption and VOC molecular size [83]. From the viewpoint of crystal structure, as a crystalline compound, MOFs have semiconductor properties because the conduction band can be composed of an outer space orbit of a metal center and the valence band can be composed of an outer orbit of an organic ligand. Under the irradiation of light, the organic ligands serving as photonic antennas will be excited to produce electrons and holes, which is the basic process of a photocatalytic reaction. Compared with the traditional photocatalyst, the structure of MOFs with large surface area and high porosity contributes to mass transfer in the photocatalytic reaction [106]. Moreover, controllable chemical and physical properties can be easily achieved by adjusting the organic functional groups and metal centers at the molecular level. Several interactions have been observed to enhance adsorption properties such as cation–π interactions, π–π interactions/stacking, hydrophobic interactions, hydrogen bonding, acid–base interactions, and pore/size selective adsorption. Wang et al. [107] studied the adsorption characteristics of MOFs for NO and NO_2_ using the Monte Carlo simulation (GCMC) method, and selected two representative MOFs (MOF-5 and MOF-177 [108]) with different topological structures, both of which were synthesized by the solvothermal method, as the models. It was found that the adsorption characteristics of MOFs were not only related to the structural characteristics of the materials themselves, but also depended on the adsorption heat.

### 4.2. Adsorption and Degradation Mechanism of MOFs

**Figure 13:**
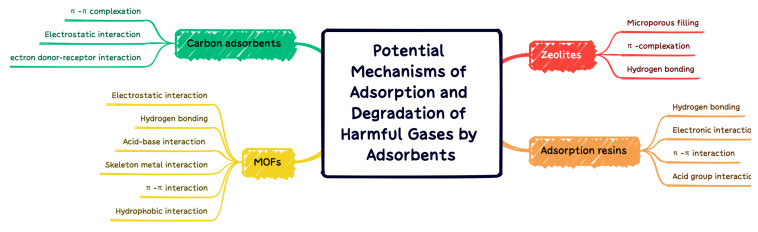
Adsorption and degradation mechanisms of carbon adsorbents, zeolites, adsorption resins, and MOFs.

MOFs exhibit high adsorption capacity for VOCs due to their high surface area, abundant functions, and abundant open metal sites. Several types of chemical bonds can be formed between VOCs and MOFs. The interaction forces can be divided into the following four aspects: (1) the adsorbate coordinates with an open metal site (coordination unsaturated metal cation); (2) adsorbates adsorbed by hydrogen bonds; (3) adsorbents are adsorbed through acid–base interaction; (4) adsorbents in the framework of MOFs. The adsorption mechanism generally involves non-specific interactions (Van Der Waals forces) and specific interactions (π-complex, H-bond, electrostatic interaction, etc.). A comprehensive experimental/computational study was conducted by Wu et al. [109]. It was found that the interaction between the conjugated π-system and thiophene sulfur atom lone pair, as well as the unsaturated site on Cu-BTC, determine the adsorption strength of thiophene compounds on Cu-BTC. Although, it is proposed that the adsorption mechanism of N compounds on MOF vary with N type, including N basicity, H substitution, and positive charge on H bonded to N. The N-coordinated MOF facilitates enhancement of the local electrostatic field, which further facilitates the adsorption of VOC with electron withdrawing groups. Hu et al. [110] found that the adsorption capacity of chlorobenzene, expressed as unit surface area, was remarkably improved after the modification of UiO-66(Zr) with dopamine compared with unmodified UiO-66(Zr). The coordination of N with the Zr cluster in UiO-66(Zr) enhances the local electrostatic field distribution and thus facilitates the adsorption of chlorobenzene with an electron-withdrawing group. For those who capture VOCs through the pore-filling mechanism, the pore size is one of the important parameters for the ability of MOF to adsorb VOCs. Yang et al. [44] demonstrated a negative linear relationship between the volumetric adsorption capacity of VOCs on MIL-101 and their molecular cross-sectional areas, demonstrating that VOCs (acetone, benzene, and toluene) had high adsorption capacity and rapid diffusion with relatively small dimensions. Hasan et al. [111] conducted a comprehensive review of possible mechanisms for the removal of contaminants by adsorption with MOFs. Electrostatic interaction, hydrogen bonding, acid–base interaction, skeleton metal interaction, π–π interaction, and hydrophobic interaction are proposed for adsorption to remove the harm of different properties. In Figure 13, the adsorption and degradation mechanisms of MOFs with carbon adsorbents, zeolites, and adsorption resins are summarized and compared.

### 4.3. Application of MOFs for VOC Removal

MOFs have been used for adsorption applications in the liquid and gas phases [112,113]. For example, Hamon et al. [113] reported the adsorption of H_2_S on various MOFs, including MIL-53 (aluminum, chromium, iron), MIL-47(V), MIL-101 (chromium), and MIL-100 (chromium). Haque et al. [114] reported a potential application of chromium-based MOF as an absorbent for the removal of methyl orange (MO). Due to the anionic nature of MO, the cationic MOFs exhibit increased adsorption capacity and rapid dye absorption. MOF-235 and MIL-100 (iron) have been used to remove the cationic methylene blue (MB) E through a similar charge mechanism. Petit et al. [115] used metal–organic frameworks (MOF; Cu-containing HKUST-1) and GO to test the adsorption of hydrogen sulfide (H_2_S) and nitrogen dioxide (NO_2_). Cho et al. [116] synthesized Co-MOF-74 by microwave heating and tested its application to CO_2_ adsorption and catalysis. The conversion of styrene oxide increased with increasing carbon dioxide pressure and reaction temperature. No significant effect of catalyst particle size was observed, and Co-MOF-74(M) could be reused three times without loss of catalytic activity and without structural deterioration. Choi et al. [117] modified Mg/DOBDC MOF with amines to enhance CO_2_ adsorption for ultra-rarefied gases. Mg/DOBDC was modified by functionalization of its open metal coordination sites. Mg/DOBDC was modified with ethylene diamine (ED) to introduce pituitary amines into the pores of the MOF. This modification not only increases the CO_2_ adsorption capacity of the material at the ultra-fine CO_2_ partial pressure, but also increases the regeneration capacity of the material. Table 2 summarizes some applications of MOFs for adsorption and degradation of VOCs.

Catalysts are important components in chemical reactions and several catalysts have been reported. MOF materials can also be used as a catalyst due to their special structure. The group of Garcia [120] investigated the performance of a highly stable Al(III) MOF called DUT-4 for SO_2_ gas adsorption. It was found that DUT-4 showed good SO_2_ capture performance, with one of the best MOFs with similar pore size volume reported so far in terms of adsorption capacity at 298 K and 1 bar, and good cyclability and easy adsorption reversibility. Furthermore, due to the high thermal stability and robust structure of DUT-4, it is also suitable for CH_4_ purification in the presence of trace amounts of SO_2_. In addition, studies on the catalytic applications of MOFs have been conducted by Fischer’s group. Hussain et al. [121] explored the visible light photocatalysis of multifunctional co-doped TiO_2_/C nanocomposites derived from metal–organic frameworks. Multifunctional and co-doped TiO_2_/C nanocomposites were obtained by pyrolysis of Ti-MOFs at 800 °C under different gas environments and their photocatalytic properties were investigated. The photocatalytic performance of the composites could be further improved by optimizing the combination of surface-attached functional groups and metal/non-metal co-dopants in TiO_2_. Li’s group [122] also developed MOF-derived highly efficient catalytic carbon-based nanomaterials. It was found that MOF-derived carbon-based nanomaterials have great advantages over other carbon-based catalysts in terms of customizable morphology, layered porosity, and easy functionalization with other heteroatoms and metals/metal oxides, which make them efficient as catalysts directly or as catalyst carriers for many important reactions. Although MOFs have many positive features in multiphase catalysis, they also have a number of drawbacks and defects. A major drawback of many (but not all) MOFs is their lack of structural stability under reaction conditions and their inability to be reactivated by thermal treatment. This leaves some MOFs that change their structure during storage due to the effects of moisture, such as MOF-5 [123].

### 4.4. The Stability of MOFs

MOFs can act as catalysts using photocatalytic or selective catalytic reactions in the presence of NH_3_, CO, or H_2_. It is to highlight high selectivity (>99%) and conversion (>95%) results obtained in the range of 200–300 °C by judiciously selecting/combining appropriate metallic cations, such as Mn^2+^, Co^2+^, Ni^2+^, and even Ce^4+^. This temperature can be further lowered if the MOF platform was optimized by adding nanoparticles within their pores. Highly stable MOF materials such as the MIL family have demonstrated superior chemical stability toward NO species, which has been confirmed by comparing X-ray diffraction (XRD) patterns of the samples before and after NO exposure [124].

Before being applied to wastewater treatment, the structure of MOFs and the stability of the ligand-metal-binding network must be studied in depth. The poor stability of MOFs in aqueous solutions and organic solvents, especially in water, severely limits their application in pollutant removal [125]. In addition, under high outturn treatment, the stability of MOFs was mainly affected by metal oxidation state and coordination strength between ligands and metal ions [126]. Studies have shown that HKUST-1 is moisture sensitive, while MIL-101, ZIF-8, and MIL-100(Fe) show high stability in direct contact with water [127,128]. Moreover, stability studies under water, steam, and about 90% humidity showed that UiO-66-NH_2_ (derivative of UiO-66 with -NH_2_ as functional group) and Mg MOF-74 exhibited high stability, while DMOF-1 and DMOF-1-NH_2_ (DMOF-1 with amine-functionalized ligands) were highly unstable [129].

## 5. Critical Assessment of Removal of VOC by MOFs

### 5.1. MOF Internal Assessment: Advantages and Disadvantages

In short, MOF is the most promising adsorbent for VOCs because of its adjustable pore structure and extraordinary physicochemical properties. In general, the adsorption capacity of MOF for VOCs is superior to that of traditional adsorbents (AC and zeolite). Modification techniques can be flexibly applied to MOF to improve hydrophobicity and adsorption selectivity. Nevertheless, there are some disadvantages in its industrial application, such as weak dispersibility due to its large void spaces and insufficient open metal sites for coordination and catalysis [130]. In addition, the use of MOF to adsorb VOCs remains an overwhelming option due to the high cost of preparation [131].

**Figure 14:**
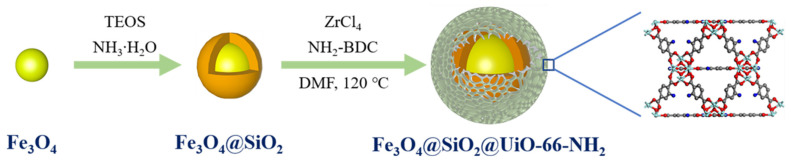
Synthesis of core-shell magnetic MOF composites [118].

Over the past few decades, MOFs have gained increasing interest in the capture and adsorption of precious metals. Chang et al. [118] prepared magnetic MOF composites with Fe_3_O_4_@SiO_2_ as the core and UiO-66-NH_2_ as the shell to adsorb Au (III) from aqueous solution (Figure 14). The underpinning adsorption mechanisms were studied through experimental characterization, molecular dynamics simulation (MDS), and density functional theory (DFT) studies. The magnetic Zr-based MOF composites exhibit great industrial value in gold recycling with high adsorption selectivity and good recyclability. El-Monaem et al. [119] found that the UiO-66/ZIF-8/PDA@CA composite beads demonstrated large specific surface area, good adsorption performance with a relatively short equilibrium time (60 min), and ease separation under optimum adsorption conditions. The thermodynamics studies found that the adsorption process was endothermic and spontaneous. Moreover, the floated UiO-66/ZIF-8/PDA@CA beads displayed better adsorption properties for eight reuse cycles with a maximal removal of 67%, reflecting its promising applicability as a reusable adsorbent for efficient removal of antibiotics from water bodies.

MOF materials have a wide range of potential applications in gas storage and separation, fuel storage, catalysis, sensing, drug transportation, and other fields due to their designable rich structures, low-density skeleton, ultra-high specific surface area, permanent pores, and functionalized pore spaces. For the practical application of MOF materials, the first consideration must be to ensure the integrity of the frame structure, so as to maintain the desired function and characteristics. The stability of MOF is affected by a variety of factors, including the operating environment, metal ions, organic ligands, metal ligand coordination geometry, and the hydrophobicity of the pore surface. Most of the early synthesized MOF materials are not suitable for practical application due to stability problems. In recent years, the problem of poor stability of MOF materials has been gradually overcome. The materials that have good stability and have been widely used mainly as MOF, ZIF, MIL, UiO, and HKUST. Based on the structure and performance characteristics of MOF materials in the field of environmental pollution control, MOF and composite MOF materials have been applied to remove a variety of pollutants and showed a broad application prospect.

### 5.2. Comparison of MOFs with Other Adsorbents

On the premise of not affecting the inherent structure of the material, the adsorption performance of the MOFs can be improved by a post-synthesis modification method [132]. Compared with other nano-catalysts, MOFs have the characteristics of excellent physical and chemical properties, adjustable pore size, diverse structures, and easy functionalization. They exhibit excellent performances in the application fields such as catalysis, adsorption, separation, and degradation, and can quickly and effectively remove various pollutants. They are more controllable and efficient in synthesis compared with conventional adsorbents. Lin et al. [133] found that the stone imidazole ester framework material (ZIF-67) had excellent adsorption performance for malachite green (MG), with an adsorption capacity (Q) as high as 2430 mg g^−1^. MOFs have adjustable pore size and electrical properties, which make it easy for them to adsorb organic molecules with the same pore size and opposite electrical properties.

**Figure 15:**
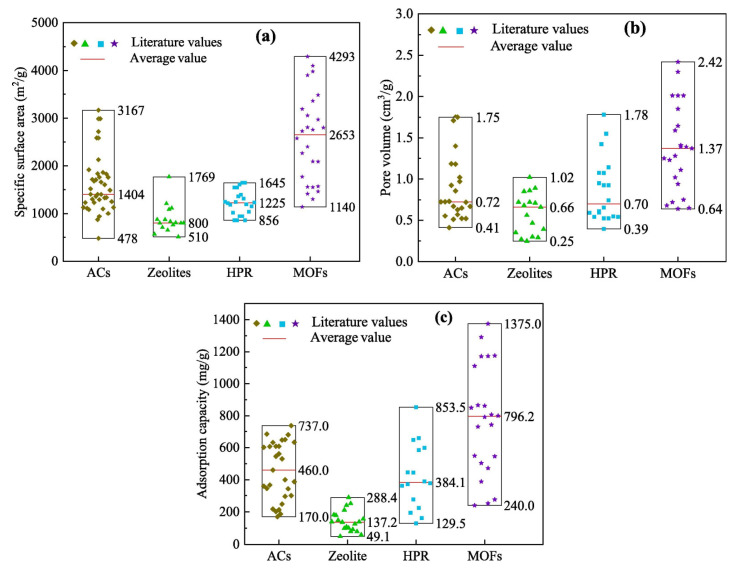
Comparison of different adsorption materials (ACs; Zeolites; HPR; MOFs): (**a**) specific surface area; (**b**) pore volume; (**c**) adsorption capacity [77].

The specific surface areas of different adsorption materials are shown in Figure 15a. Compared with other adsorption materials, MOF has the largest specific surface area. The average specific surface area of MOFs is 2653 m^2^/g, with a maximum of 4293 m^2^/g, which is 1.89 times that of activated carbon, 3.32 times that of zeolite, and 2.16 times that of HPR. The pore volumes of the different adsorbents are shown in Figure 15b. The average pore volume of the organometallic framework is 1.37 cm^3^/g, with a maximum of 2.42 cm^3^/g. The comparison of the adsorption capacity of VOCs on different adsorption materials is shown in Figure 15c. The average adsorption capacity of the organometallic framework was 796.2 mg/g, with a maximum adsorption capacity of 1375.0 mg/g. Clearly, MOFs have the best adsorption capacity compared to activated carbon, zeolites, and crosslinked polymeric resins. The adsorption capacity of MOFs for VOCs is 1.7 times that of activated carbon, and 5.8 times and 2.1 times that of zeolite and super crosslinked polymer resin, respectively [77].

## 6. Conclusions, Perspectives, and Future Perspectives

VOCs are a major threat to human life and health. The technologies currently used to remove VOCs mainly include adsorption and photocatalysis. Adsorption is the most straightforward strategy, but it cannot ultimately eliminate VOCs. Due to the limited binding surface, the formaldehyde adsorption on conventional photocatalysts is limited and the photocatalytic degradation efficiency is not high enough. Compared with non-porous photocatalysts, the porous structure in photocatalysts can not only improve the adsorption of formaldehyde, but also improve the photocatalytic efficiency due to more abundant active chemical sites and shorter electron transfer distances, while promoting mass diffusion and improving light transmission. Hierarchical porous structures with high efficiency are more attractive, so porous photocatalysts are desirable for the elimination of VOCs.

The unique physicochemical properties of MOF materials, such as huge specific surface area, tunable pores, excellent photogenerated electron transport ability, structural ordering, easy modification, etc., especially their high designability at the molecular level, make them ideal for a class of microporous/mesoporous materials that has developed rapidly in the past decade. However, compared with many scientific research achievements reported so far, the MOF materials that can truly achieve high-efficiency and high-selectivity catalytic effects are still very limited, and the photocatalytic process needs to rely on sacrificial agents to achieve better catalytic effects. It is neither environmentally friendly nor economical, and also has great restrictions on the practical application of catalysts. To achieve efficient photocatalysis, it is urgent to develop a photocatalyst that can respond to visible light. Therefore, the development and expansion of the types of MOF-based photocatalytic materials creates more opportunities for the research and development of independent and efficient MOF materials. In addition, the morphological design and surface microstructure control of MOFs are of great significance for exploring the crystal growth mechanism and developing novel nanomaterials with excellent performance and application prospects.

Although there are still many challenges in the construction of multifunctional MOF-based photocatalytic materials, the promising development prospects of new functional materials and the increasing research in this field indicate the vigorous development potential of MOF materials in the field of photocatalysis.

## Figures and Tables

**Table 1:** The VOCs are classified by boiling point according to WHO.

Boiling Point	Name	Examples of VOCs and Boiling Points
Boiling point < 50 °C	Very volatile organic compounds (VVOCs)	Methane (−161 °C), formaldehyde (−21 °C), methyl mercaptan (6 °C), acetaldehyde (20 °C), methylene chloride (40 °C)
50 °C ≤ Boiling point < 260 °C	Volatile organic compounds (VOCs)	Ethyl acetate (77 °C), ethanol (78 °C), benzene (80 °C), methyl ethyl ketone (80 °C), toluene (110 °C), trichloroethane (113 °C), xylene (140 °C), Boehmeria (178 °C), nicotine (247 °C)
260 °C ≤ Boiling point < 400 °C	Semi-volatile organic compounds (SVOCs)	Chlorpyrifos (290 °C), dibutyl phthalate (340 °C), di(2-hexyl) phthalate (390 °C)
400 °C ≤ Boiling point	Granular organic compounds (POMs)	Polychlorinated biphenyl (PCB) benzo(a)pyrene

**Table 2:** Applications of MOFs for adsorption and degradation of VOCs.

MOF Adsorbents	Applications	Refs.
MIL-53 (aluminum, chromium, iron)	Remove harmful gases	[113]
MIL-47 (V)		
MIL-101 (chromium)		
MIL-100 (chromium)		
Chromium-based MOF	Removal of methyl orange	[114]
MOF-235 and MIL-100 (iron)	Remove the cationic methylene blue	[114]
Cu-containing HKUST-1	The adsorption of H_2_S and NO_2_	[115]
Co-MOF-74	CO_2_ adsorption and catalysis	[116]
Magnetic Zr-based MOF composites	Capture and adsorption of precious metals	[118]
UiO-66/ZIF-8/PDA@CA	The adsorptive removal of tetracycline (TC)	[119]

## Data Availability

Not applicable.
